# IL-32θ gene expression in acute myeloid leukemia suppresses TNF-α production

**DOI:** 10.18632/oncotarget.5688

**Published:** 2015-10-16

**Authors:** Man Sub Kim, Jeong-Woo Kang, Jae-Sik Jeon, Jae Kyung Kim, Jong Wan Kim, Jintae Hong, Do-Young Yoon

**Affiliations:** ^1^ Department of Bioscience and Biotechnology, Bio/Molecular Informatics Center, Konkuk University, Seoul, Republic of Korea; ^2^ Department of Laboratory Medicine, Dankook University College of Medicine, Cheonan, Korea; ^3^ Dankook University College of Health Sciences, Department of Biomedical Laboratory Science, Cheonan, Korea; ^4^ College of Pharmacy, Medical Research Center, Chungbuk National University, Chungbuk, Korea; ^5^ Current address: Seegene Inc., Seoul, Korea

**Keywords:** IL-32, TNF-α, acute myeloid leukemia (AML), NF-κB

## Abstract

The proinflammatory cytokine TNF-α is highly expressed in patients with acute myeloid leukemia (AML) and has been demonstrated to induce rapid proliferation of leukemic blasts. Thus suppressing the production of TNF-α is important because TNF-α can auto-regulate own expression through activation of NF-κB and p38 mitogen-activated protein kinase (MAPK). In this study, we focused on the inhibitory effect of IL-32θ on TNF-α production in acute myeloid leukemia. Approximately 38% of patients with AML express endogenous IL-32θ, which is not expressed in healthy individuals. Furthermore, plasma samples were classified into groups with or without IL-32θ; then, we measured proinflammatory cytokine TNF-α, IL-1β, and IL-6 levels. TNF-α production was not increased in patients with IL-32θ expression than that in the no-IL-32θ group. Using an IL-32θ stable expression system in leukemia cell lines, we found that IL-32θ attenuated phorbol 12-myristate 13-acetate (PMA)-induced TNF-α production. IL-32θ inhibited phosphorylation of p38 MAPK, inhibitor of κB (IκB), and nuclear factor κB (NF-κB), which are key positive regulators of TNF-α expression, and inhibited nuclear translocation of NF-κB. Moreover, the presence of IL-32θ attenuated TNF-α promoter activity and the binding of NF-κB with the TNF-α promoter. In addition, IL-32γ-induced TNF-α production has no correlation with inhibition of TNF-α via IL-32θ expression. Thus, IL-32θ may serve as a potent inhibitor of TNF-α in patients with AML.

## INTRODUCTION

Tumor necrosis factor α (TNF-α), a key proinflammatory cytokine, is known to be implicated in the pathogenesis of some diseases [1] and is predominantly expressed by activated macrophages, monocytes, T lymphocytes, and other cell types [2–5]. TNF-α is cleaved as a 26-kDa protein in the extracellular domain by a converting enzyme and subsequently secreted as a soluble trimeric complex [6]. TNF-α can activate target cells by interaction with 1 of 2 receptors, TNFR1 and TNFR2, which are individually regulated depending on various cell types in healthy and diseased tissues [7]. Dysregulation of TNF-α results in the onset of diseases such as rheumatoid arthritis (RA) [8–9], inflammatory bowel disease (IBD) [10–12], Alzheimer's disease [13–14], and some cancers [15–16]. Accordingly, the expression of TNF-α is tightly regulated during an immune response to an infection or during autoimmune diseases, and there are many drugs targeting TNF-α and its receptors, for example, infliximab, certolizumab, and etanercept, which are designed for the treatment of inflammatory diseases such as rheumatoid arthritis [17–18]. Acute myeloid leukemia (AML) is a cancer affecting hematopoietic stem cells, where accumulation of dysfunctional myeloid cells is accompanied by the reduced production of healthy blood cells [19]. AML is caused by various factors such as mutations [20–21], radiation [22], and carcinogens [23]. AML is closely associated with cytokine networks in terms of proliferation, apoptosis, and differentiation of leukemic cells [24]. Patients with AML maintain expression of various cytokines at levels higher than those in healthy individuals The production of cytokines such as TNF-α, IL-6, and IL-1β, that are driven during a leukemogenesis, stimulates AML blast growth *in vitro* via colony stimulating factor (CSF)-induced clonogenicity [25]; on the other hand, IL-10 downregulates cytokines that are involved in the differentiation and proliferation of AML cells [26]. Previous clinical studies with AML patients have confirmed that the NF-κB is constitutively active and maintained in the form of RelA/p50 and p50/p50 complexes [27]. Especially, high level of TNF-α expression is kept consistently by NF-κB activation in AML blasts, and results in persisting proliferation [28].

IL-32 was recently identified as a TNF-α inducer for the first time [29]. IL-32 is also mainly involved in major inflammatory diseases such as RA [30–31], IBD [32], and chronic obstructive pulmonary disease (COPD) [33]. There are 8 exons in the IL-32 gene. In addition to the existing 5 splicing variants (IL-32α, IL-32β, IL-32δ, IL-32ε, and IL-32ζ) from IL-32γ, three isoforms (IL-32θ, IL-32θ, and IL-32s) were uncovered in our previous study [34]. Several transcriptional variants of IL-32 play a pivotal role in inflammation by inducing proinflammatory cytokines such as TNF-α, IL-1β, and IL-6 in patients with inflammatory disorders and in cell-based models [35–36]. IL-32 plays various roles in cancer biology such as cancer cell survival as well as apoptotic death in accordance with each IL-32 isoforms. IL-32γ inhibits cancer cell growth via inactivation of NF-κB and STAT3 in colon cancer [37]. IL-32α showed the apoptotic killing in colorectal cancer through TNFR-1 mediated signaling [38]. In addition, IL-32β is associated with enhancement of cancer cell growth and invasion [39–40]. However, the intracellular role of IL-32 has been recently investigated that IL-32 binds to protein kinase *c* (PKC) isoforms and thereby regulates followed signaling pathway [41].

The aim of the present study was to determine the possible pathological function of IL-32θ in patients with AML. We also analyzed the mechanism of TNF-α downregulation via suppression of p38 MAPK and NF-κB activities in phorbol 12-myristate 13-acetate (PMA)-activated leukemia cell lines. This study could provide pivotal evidence of the role of IL-32θ in hematological diseases.

## RESULTS

### IL-32θ mRNA detected in patients with acute myeloid leukemia

**Figure 1:**
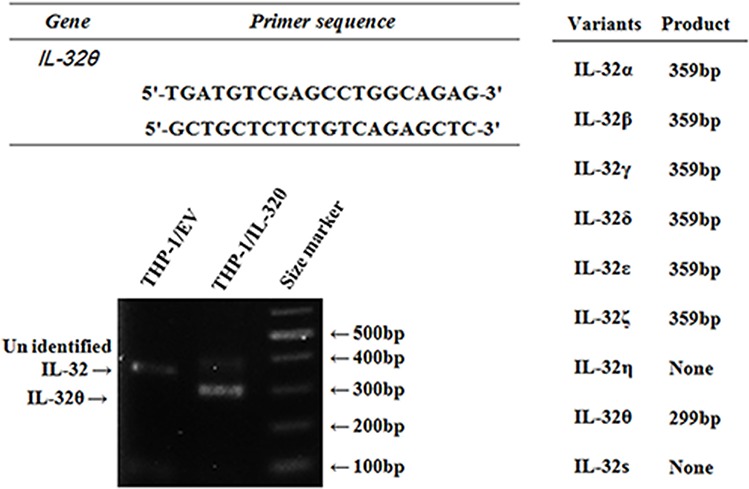
The design of specific primers which distinguish endogenous IL-32θ among various isoforms The expected PCR product size (when using specific primers) of each isoform is described. IL-32θ-specific primers produce an IL-32θ band that is different from the bands of other IL-32 isoforms. The IL-32θ fragment was amplified (by means of specific primers) from THP-1 cells stably expressing IL-32θ.

IL-32 gene has nine isoforms because of alternative mRNA splicing [45]. Existing primers for identification of each isoform were problematic for our purposes. We first designed forward and reverse primers specific to IL-32θ to determine the expression levels of endogenous IL-32θ in comparison with the other splicing variants. IL-32θ lacks exon 6 in mRNA, unlike the other IL-32 isoforms; thus, we designed the forward and reverse primers so that they bind to exon 5 and exon 7. With the help of these primers, IL-32θ and the other isoforms were distinguished based on the differences in the size of amplicons: the size of the IL-32θ PCR product was 299 bp while the other isoforms yielded amplicons of 359 bp. Thus, we confirmed the validity of these primers on THP-1 cell clones stably expressing the empty vector or IL-32θ (Figure 1). To test whether the sequence of the amplicon in THP-1/IL-32θ cells matched that of the IL-32θ, we analyzed the sequence of the PCR amplicon and compared it to the IL-32θ mRNA sequence. The PCR product perfectly matched IL-32θ (data not shown).

### IL-32θ negatively regulates TNF-α production in AML patients

**Figure 2:**
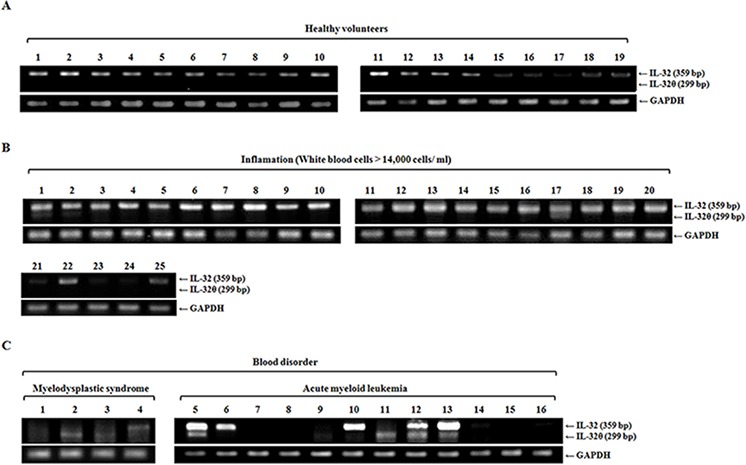

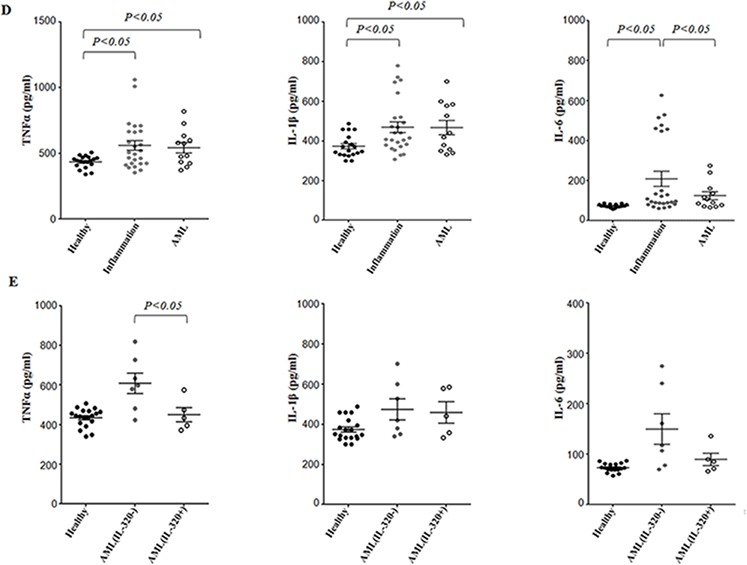
Inhibitory effects of IL-32θ on the production TNF-α in AML IL-32θ expression levels were measured by RT-PCR using blood samples from healthy individuals **A.** and from patients with a severe inflammatory disease **B.** or blood disorders **C.** such as myelodysplastic syndrome (MDS) and acute myeloid leukemia (AML). cDNA was prepared from 1-mL blood samples, and each cDNA sample was analyzed by PCR with IL-32θ primers. **D.** and **E.** The levels of TNF-α, IL-1β, and IL-6 were measured by an enzyme-linked immunosorbent assay (ELISA) of plasma prepared from the same blood samples. The data are presented as mean ± standard deviation (*n* = 3); *P < 0.05.

We first confirmed endogenous IL-32θ expression in healthy volunteers and in patients with a high level of white blood cells. In the group of healthy individuals (*n* = 20), IL-32θ was not detectable, whereas other IL-32 isotypes were expressed according to our specific primers (Figure 2A). In contrast, as shown in Figures 2B and 2C, IL-32θ was noticeably expressed in AML patients (5/13) and patients with myelodysplastic syndrome (MDS) (2/4), at a level higher than that in patients with a severe inflammatory disease (2/24). To test whether IL-32θ expression suppresses proinflammatory cytokine production in AML, we first measured the production of TNF-α, IL-1β, and IL-6 in patients with AML compared the levels to that found in healthy individuals and patients with a severe inflammatory disease (Figure 2D). The production of TNF-α and IL-1β was higher in AML patients than in healthy individuals, but not in patients with a severe inflammatory disease. There was no significant difference in IL-6 production between healthy individuals and patients with AML. We next conducted comparative analysis: the patients with AML were subdivided into the group with IL-32θ expression and the group without (Figure 2E). In patients with AML, the group expressing IL-32θ showed significantly decreased production of TNF-α. This finding suggested that IL-32θ expression was inducible under specific conditions and could control TNF-α production in AML patients.

### IL-32θ attenuates induction of TNF-α production by phorbol ester

**Figure 3:**
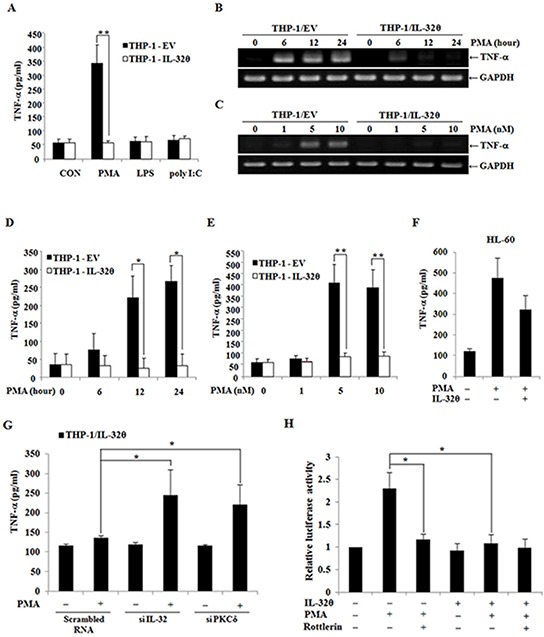
Inhibitory effects of IL-32θ on the production TNF-α in THP-1 cells **A.** THP-1/EV and THP-1/IL-32θ cells were treated with phorbol 12-myristate 13-acetate (PMA), lipopolysaccharide (LPS), and poly(I:C) for 24 h to activate THP-1 cells. TNF-α production was assessed by ELISA in the culture supernatants after the above stimuli. The cells were incubated with 10 nM PMA and were analyzed at different time points **B, D.** alternatively, different doses of PMA were used for 24 h **C, E.** After that, TNF-α mRNA levels were assessed by RT-PCR, and GAPDH was used as an internal control. The secreted TNF-α protein was also quantified by ELISA in the culture supernatants. **F.** HL-60 cells were transfected with 1 μg of pcDNA3.1+-6xMyc and 6xMyc-IL-32θ and were treated with PMA. The secreted TNF-α protein was also quantified by ELISA in the culture supernatants. **G.** THP-1-IL-32θ cells were transfected with 100 nM scrambled siRNA and siRNA against IL-32θ and then, PKCδ and TNF-α secretion was measured. The secretion levels of TNF-α were measured by an ELISA in culture supernatants after incubation of the cells with 10 nM PMA for 24 h. **H.** The structure of the TNF-α promoter between positions −1236 and +28. THP-1 cells were transfected with 1 μg of TNF-α-promoter-Luc and an IL-32θ expression vector overnight. Rottlerin (10 μM) was added to the culture medium 1 h before 10 nM PMA treatment. Promoter activity was measured by means of a dual-luciferase reporter assay system. The data are presented as mean ± standard deviation (*n* = 3); *P < 0.05.

To determine how IL-32θ attenuates TNF-α production in the cells, we utilized a previously established IL-32θ stable expression system in THP-1 human monocytes; these cells express little or no IL-32θ endogenously [42]. Even though THP-1 monocytes are derived from a patient with leukemia, these cells need specific stimulation for them to mimic AML-like properties because typical AML blasts are activated by such signals as PKC [46], which is not the case for THP-1 cell lines. Thus, we next administered several external stimuli such as PMA, LPS, and poly(I:C) to induce TNF-α production in THP-1/EV and THP-1/IL-32θ cells. Only PMA treatment significantly increased TNF-α production in THP-1/EV cells (Figure 3A). Moreover, we measured the mRNA expression and secretion of TNF-α by RT-PCR and ELISA, and the results showed that TNF-α production was enhanced by PMA in a time- and dose-dependent manner in THP-1/EV cells but not in THP-1/IL-32θ cells (Figure 3B, 3C 3D, and 3E). Additionally, human promyelocytic HL-60 cells were treated with PMA after transfection with 1 μg of IL-32θ to induce TNF-α production and then we measured cytokine level by ELISA. TNF-α production is partially decreased in IL-32θ-transfected cells (Figure 3F). These findings indicated that IL-32θ attenuated PMA-induced TNF-α production in human myeloid leukemia cells.

We next transfected siRNAs specific to IL-32θ and PKCδ into THP-1/IL-32θ cells, and then we performed comparative analysis of TNF-α production with or without PMA stimulation. The IL-32θ-mediated suppression of TNF-α production was reversed by siRNA against IL-32θ or PKCδ (Figure 3G). Furthermore, we next measured TNF-α promoter activity to determine whether the downregulation of TNF-α expression by IL-32θ involved PKCδ because we previously studied the association of IL-32θ and PKCδ [33]. In the presence of IL-32θ, TNF-α promoter activity was decreased even when the cells were treated with PMA (Figure 3H). These findings indicated that IL-32θ inhibited TNF-α production via PKC signaling pathways.

### IL-32θ inhibits phosphorylation of p38 MAPK

**Figure 4:**
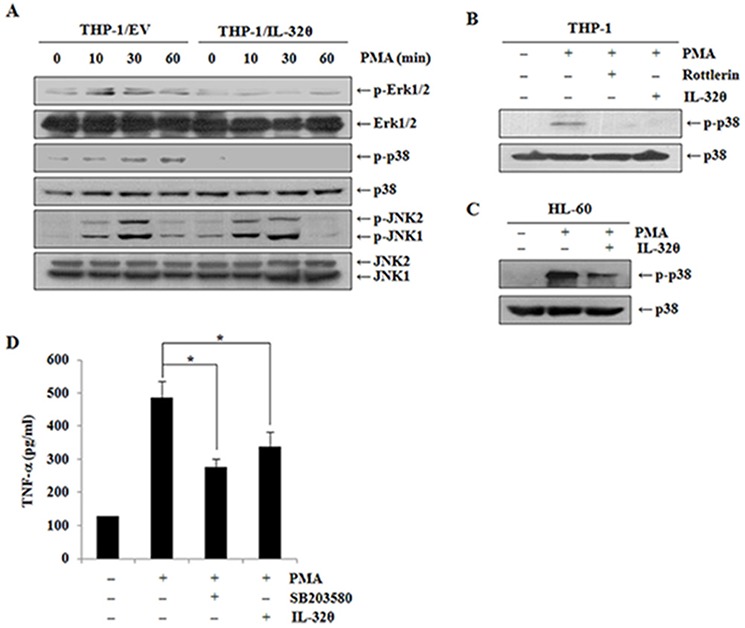
Inhibitory effects of IL-32θ on phorbol 12-myristate 13-acetate (PMA)-mediated p38 MAPK activation **A.** THP-1/EV cells and THP-1/IL-32θ cells were incubated with 10 nM PMA at the indicated time points. The activation of Erk, p38, and JNK MAPK was quantified by Western blotting. **B.** THP-1 cells were transfected with 1 μg of pcDNA3.1+-6xMyc and 6xMyc-IL-32θ and were pretreated with rottlerin before PMA stimulation. The activation of p38 MAPK was assessed by Western blotting. **C.** HL-60 cells were transfected with 1 μg of pcDNA3.1+-6xMyc and 6xMyc-IL-32θ and were treated with PMA. The activation of p38 MAPK was assessed by Western blotting. **D.** THP-1 cells were transfected with 1 μg of pcDNA3.1+-6xMyc and 6xMyc-IL-32θ and were pretreated with SB203580 before PMA stimulation. The secretion levels of TNF-α were measured by an ELISA in culture supernatants. The data are presented as mean ± standard deviation (*n* = 3); *P < 0.05.

To determine which signaling pathways influence TNF-α production during PMA treatment, we first examined phosphorylation of MAPKs such as Erk, p38, and JNK, which are major regulators of TNF-α expression. We used 10 nM PMA at various time points to test phosphorylation of the intracellular kinases. As shown in Figure 4A, phosphorylation levels of p38 are clearly attenuated in THP-1/IL-32θ cells than that in the control cells. IL-32 is known to interact with PKC resulting in regulation of phosphorylation of PKC substrates [42, 47–48]. Thus, we next tested the association of PKCδ with IL-32θ in terms of p38 MAPK phosphorylation (Figure 4B). Activation of p38 MAPK was significantly suppressed by rottlerin, a PKCδ-specific inhibitor, and by IL-32θ. After HL-60 cells were transiently transfected with 1 μg of IL-32θ, we confirmed whether p38 MAPK was inhibited by IL-32θ expression in HL-60 myelocyte. The transfection of IL-32θ resulted in partial inhibition of p38 phosphorylation (Figure 4C). Moreover, IL-32θ as well as SB203580, a p38 specific inhibitor, were involved in suppression of PMA-induced TNF-α production (Figure 4D). These findings seem to reveal the molecular mechanisms underlying the effects of IL-32θ on TNF-α expression.

### NF-κB activation and nuclear translocation are inhibited by IL-32θ

**Figure 5:**
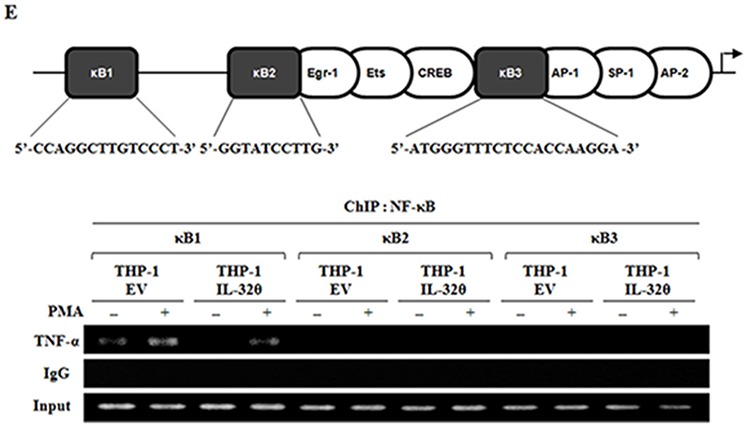

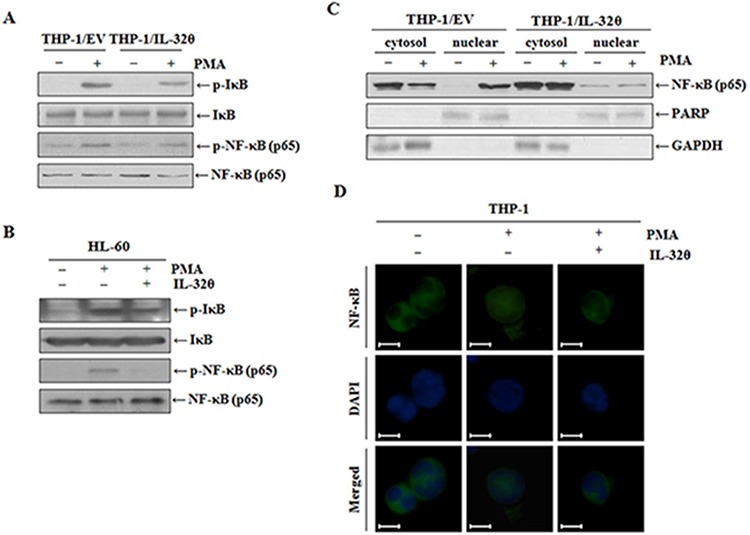
IL-32θ suppressed NF-κB activation, resulting in the disruption of NF-κB's binding to the TNF-α promoter at the κB1 site **A.** THP-1/EV and THP-1/IL-32θ cells were incubated with 10 nM PMA for 1 h. The phosphorylation levels of IκB and NF-κB were assessed by Western blotting. **B.** HL-60 cells were transfected with 1 μg of pcDNA3.1+-6xMyc and 6xMyc-IL-32θ and were treated with 10 nM of PMA for 1 h. The phosphorylation levels of IκB and NF-κB were assessed by Western blotting. **C.** THP-1/EV and THP-1/IL-32θ cells were fractionated into the nuclear and cytosolic parts, and were then stimulated with 10 nM PMA. Nuclear translocation of NF-κB was confirmed by Western blotting. GAPDH and PARP served as controls in the cytosol and nucleus, respectively. **D.** NF-κB nuclear translocation was assessed by an immunofluorescence assay. THP-1 cells were transfected with 2 μg of pcDNA3.1+-6xMyc and 6xMyc-IL-32θ overnight and were incubated with 10 nM PMA. After fixing with acetone, we stained NF-κB with the relevant primary antibodies and secondary antibodies conjugated with FITC (green). DAPI was used for staining nuclei to visualize DNA. Scale bars represent 5 μm. **E.** A schematic showing the TNF-α promoter and the target sequences of NF-κB binding tested in our chromatin immunoprecipitation (ChIP) assays. THP-1-EV and THP-1-IL-32θ cells were incubated with 10 nM PMA for 12 h, then the cell lysates were fixed with 1% formaldehyde. The ChIP assay was carried out with 2 μg of an anti-p65 antibody, then the TNF-α promoter immunoprecipitated via NF-κB was analyzed using primers specific to the DNA consensus sequences of NF-κB-binding sites in the TNF-α promoter.

NF-κB is a key transcription factor involved in production of TNF-α in leukemia. Thus, we first examined phosphorylation of IκB, which is a cellular inhibitor of NF-κB. The phosphorylation of IκB was induced by PMA treatment in a time-dependent manner, but in the presence of IL-32θ, phospho-IκB was relatively downregulated. Accordingly, we analyzed phospho-NF-κB, and as expected, it was downregulated by IL-32θ (Figure 5A). Moreover, the same results were also obtained in HL-60 cells (Figure 5B). After transfection with 1 μg of IL-32θ, we analyzed the expression levels of each phosphorylated form. The expression of p-NF-κB and *p*-IκB was slightly suppressed by IL-32θ. The inhibition of NF-κB phosphorylation by IL-32θ disrupted its translocation into the nucleus (Figure 5C). In the fluorimetric experiment, NF-κB was translocated into the nucleus after PMA stimulation. In contrast, in the presence of IL-32θ, the nuclear translocation of NF-κB was blocked (Figure 5D). Moreover, we evaluated the NF-κB binding to the TNF-α promoter at 3 κB sites, κB1, κB2, and κB3, by means of ChIP analysis. NF-κB bound to the κB1 site during PMA stimulation but not to the other κB sites. Nonetheless, NF-κB binding to the κB1 site was attenuated in THP-1/IL-32θ cells despite PMA stimulation (Figure 5E). These findings indicated that IL-32θ suppressed NF-κB activity and caused downregulation of TNF-α gene expression.

### Anti-inflammatory effect of IL-32θ has no correlation with IL-32γ-dependent inflammation

**Figure 6:**
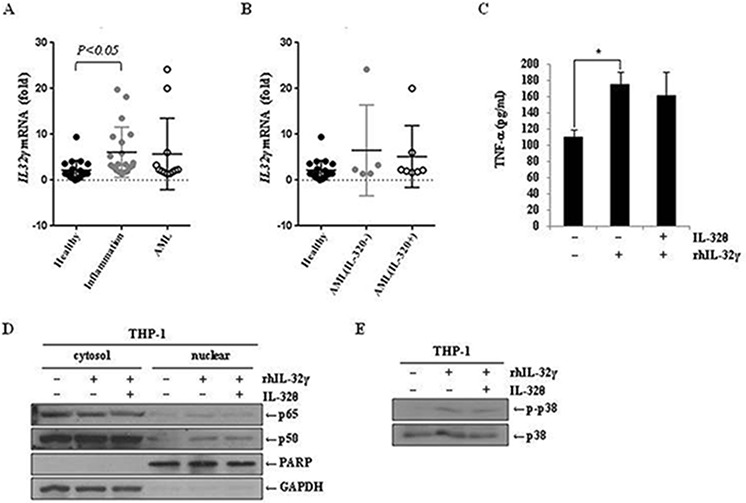
IL-32γ induced TNF-α production independent on IL-32θ signaling **A.** and **B.** IL-32γ expression levels were measured by qRT-PCR using blood samples from healthy individuals and patients with a severe inflammatory disease or blood disorders. cDNA was prepared from 1-mL blood samples, and each cDNA sample was analyzed by qPCR with IL-32γ primers. **C.** TNF-α production was measured by an ELISA from the IL-32γ treated cells. The differentiated THP-1 cells by pre-treatment of PMA were treated with 200 ng/ml of rhIL-32γ and supernatants were collected and used for ELISA assay. The data are presented as mean ± standard deviation (*n* = 3); *P < 0.05. **D.** After transfection of 1 μg of pcDNA3.1+-6xMyc and 6xMyc-IL-32θ, differentiated THP-1 cells were treated with 200 ng/ml of rhIL-32γ. The nuclear proteins were separated from cytosol, and nuclear translocation of p65 and p50 was measured by Western blot. **E.** The differentiated THP-1 cells were treated with 200 ng/ml of IL-32γ for 1 h and then the phosphorylation of p38 was measured by Western blot.

**Figure 7:**
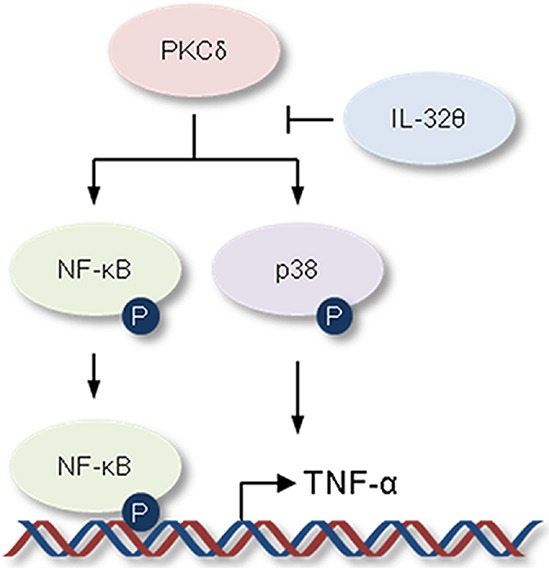
Schematic illustration showing the inhibitory effect of IL-32θ on TNF-α production The activation of p38 MAPK and NF-κB is steadily maintained by activated PKC or extrinsic cytokine-mediated receptor signaling pathway in AML. IL-32θ inhibits phosphorylations of p38 MAPK and NF-κB mediated via PKC, resulting in suppression of TNF-α production.

IL-32γ is most active isoform [49] and is well known to be a pro-inflammatory cytokine in autoimmune disease models such as rheumatoid arthritis [31]. IL-32γ can induce TNF-α production by activating NF-κB (p50) and MAPK signaling pathways [50–53]. For that reason, to confirm the association of IL-32γ signaling with the effect of IL-32θ, we first measured IL-32γ expression in healthy volunteers and patients with potential inflammation and blood disorder. IL-32γ expression was increased in both groups of patients compared to healthy individuals (Figure 6A). However, there was no change of IL-32γ expression between IL-32θ-expressing group and non-expressing group (Figure 6B). Significantly, IL-32θ had no effect on IL-32γ-induced TNF-α production in differentiated THP-1 cell line (Figure 6C). We next confirmed the translocation of NF-κB (p50 and p65) into nuclear by the cell fractionation of nuclear and cytosol in differentiated THP-1 cells. The expression levels of p65 and p50 were increased by treatment of rhIL-32γ in nuclear. However, the expression of NF-κB p50 and p65 was not altered in nuclear even though IL-32θ was expressed by transient transfection (Figure 6D). Moreover, IL-32γ-dependent phosphorylation of p38 MAPK was not altered by IL-32θ (Figure 6E). These findings showed that IL-32θ inhibits TNF-α production in an IL-32γ-independent manner. In conclusion, IL-32θ inhibited TNF-α production by suppressing the activation of p38 MAPK and p65 NF-κB, as schematically illustrated in figure 7.

## DISCUSSION

The expression and maturation of cytokines are often altered hematologic diseases including AML [54–55]. In particular, leukemia blasts of patients with AML show higher TNF-α production than do the corresponding cells in healthy individuals [56]. TNF-α plays a pleiotropic role in hematologic cancers. For example, TNF-α is expressed at a high level by AML blasts and mainly enhances production of the cytokines IL-1β and GM-CSF in terms of leukemia growth control [57]; other studies demonstrated that TNF-α can induce apoptosis in leukemic cells [58]. Furthermore, TNF-α induces senescence and chromosomal instability via a prolonged growth arrest and telomeric disturbances (shortening, loss, or fusion) [59].

In the complex molecular mechanisms of AML, typical leukemic blasts overexpress PKCs and highly activated PKCs [46]. PKC activity controls TNF-α gene transcription via PKC signaling pathways in various cell types [60]. A PKCδ-specific inhibitor, rottlerin, affects cytokine synthesis and suppresses production of TNF-α [61]. Recently, various trials were designed and conducted to prevent AML tumorigenesis by targeting PKC-mediated signal transduction pathways [62]. Most PKCs indirectly or directly activate p38 MAPK and NF-κB signaling [63–65]. In Mo7e human megakaryoblastic leukemia cell line, the activation of p38 partially supports transcriptional activation function of NF-κB (p65) [66]. Moreover, p38 can regulate NF-κB transcriptional activation leading to production of TNF-α through p65 phosphorylation [67]. Many molecules that are involved in PKC signaling pathways can serve as positive regulators of TNF-α gene expression regardless of the relation of p38 with NF-κB [68]. One study showed that NF-κB is constitutively activated through positive feedback via autocrine TNF-α secretion in AML [28]. These observations suggest that the signaling pathways that induce TNF-α production are complicated and may require activation of PKC, p38 MAPK, and NF-κB either together or separately.

IL-32 isoforms are implicated in inflammatory diseases involving upregulation of proinflammatory cytokines [29]. On the other hand, recent studies showed that IL-32 may act as an intracellular intermediary through interaction with other molecules [69–71]. Previously, we also explored the functions of IL-32 in the regulation of pro- and anti-inflammatory cytokines (such as IL-1β, IL-6, and IL-10) that are associated with PKC isoforms in the cells [42, 48, 72]. In particular, IL-32θ is considered an anti-inflammatory factor because it leads to inhibition of the expression of IL-1β and CCL-5, which are involved in the pathogenesis of inflammatory diseases via the association with PKCδ and regulation of its downstream signals. In addition, IL-32θ is associated with the suppression of monocytic differentiation into macrophage by attenuating PU.1 expression [73].

In the present study, we showed for the first time the endogenous expression of IL-32θ in AML and demonstrated that IL-32θ downregulates TNF-α gene expression in patients with AML and *in vitro*. IL-32θ is expressed in AML patients and MDS patients, and patients with AML who express IL-32θ show suppressed TNF-α production. As for the molecular mechanism, IL-32θ appears to impede TNF-α gene expression, which is involved in PKC signaling pathways including p38 MAPK and NF-κB in THP-1 cells activated by PMA treatment. Those regulators of TNF-α production are not phosphorylated in THP-1/IL-32θ cells, even if such cells are stimulated with PMA to promote PKC activity. Moreover, IL-32θ seems to block nuclear translocation of NF-κB, resulting in inhibition of the binding of NF-κB to the κB1 site in the TNF-α promoter. These findings suggest that IL-32θ expression is inducible depending on the microenvironment, and that IL-32θ may be useful for manipulation of cytokine profiles in AML.

## MATERIALS AND METHODS

### Patients with acute myeloid leukemia and healthy volunteers

Samples of venous blood (1–3 mL) were obtained from patients newly diagnosed with a potent inflammatory disease (total white-blood-cell counts > 14,000 cells/mL) or acute myeloid leukemia. The protocol of this study was approved by the Institutional Review Board (decision # 2014-03-002-001) of the Dankook University Hospital (Cheonan, Korea) (Table 1). The collected whole-blood samples were stored at 4°C and then nucleic-acid extraction was attempted within 24 h. RNA was extracted from a 200 μL of EDTA-treated whole blood using the QIAamp RNA Blood Mini Kit (Qiagen, Germany). cDNA was synthesized from the extracted RNA using the RevertAid First-Strand cDNA Synthesis Kit (Fermentas, Canada).

**Table 1:** Characteristics of the healthy individuals and patients

Group	Diagnosis	Number	Sex		Age (median)
			Male	Female	
1	Healthy individuals	19	14	5	28–75 (50.6)
2	Acute myeloid leukemia (AML)	12	11	1	15–79 (20.3)
3	Myelodysplastic syndrome (MDS)	4	4	0	68 (68)
4	Patients with severe inflammation (white blood cell count <14,000 cell/mL)	25	12	13	27–84 (49.9)

### Cell culture and establishment of cell lines with stable expression of a transgene

Human monocytic THP-1 and HL-60 cell lines were cultured in the RPMI 1640 medium (WelGENE, Taegu, Korea) supplemented with 2 mM L-glutamine, 100 U/mL penicillin, 100 μg/mL streptomycin, and 10% fetal bovine serum (HyClone, Logan, UT). The establishment of the THP-1/EV and THP-1/IL-32θ cell lines was described previously [42]. The THP-1 cells were differentiated to macrophage-like cells by treatment with PMA as previously described [43]. The HEK (human embryonic kidney) 293 cell line was grown in the DMEM medium supplemented with 2 mM L-glutamine, 100 U/mL penicillin, 100 μg/mL streptomycin, and 10% fetal bovine serum.

### Reagents and antibodies

PMA, lipopolysaccharide (LPS), and poly(I:C) were purchased from Sigma-Aldrich (St. Louis, MO). Rottlerin, a PKCδ inhibitor, and SB203580, a p38 MAPK inhibitor, were purchased from Calbiochem (San Diego, CA). The antibody specific to the myc tag was purchased from Millipore (Millipore, Bedford, MA). Antibodies specific to IκB, phospho-IκB, NF-κB (p65), phospho-NF-κB (phospho-p65), phospho-JNK, and GAPDH were purchased from Cell Signaling Technology (Beverly, MA), and antibodies to NF-κB (p50), p38, phospho-p38, Erk, phospho-Erk, and JNK were purchased from Santa Cruz Biotechnology (Santa Cruz, CA). KU32–52, a monoclonal anti-IL-32 antibody, was produced as previously reported [44]. The recombinant human IL-32γ (rhIL-32γ) was obtained from YbdY incorporation (Seoul, Korea). The siRNAs specific to human IL-32 and PKCδ were purchased from Dharmacon (Lafayette, CO).

### Construction of the expression vectors and Luc reporter plasmid for the TNF-α promoter

Subcloning of pcDNA3.1+-6xMyc-IL-32θ was described previously [42], and pcDNA3.1+5xFLAG-IL-32θ was designed using the same method. The TNF-α promoter region (positions −1236 to +28) was amplified from human genomic DNA (THP-1 cells). The primer set was 5′-GCT GTC TGC TTG TGT GTG TG-3′ (sense) and 5′-TGT CCT TGC TGA GGG AGC GT-3′ (antisense). The PCR product was digested with the *Kpn*I and *Bgl*II restriction enzymes. The digested fragment was ligated into the pGL3- Basic vector.

### Real time PCR and reverse transcription PCR assay

To detect endogenous IL-32θ mRNA expression, specific primers to distinguish IL-32θ from other isoforms were designed based on the differences in size (gene structure) among the IL-32 isoforms. The mRNA expression level of endogenous IL-32θ was detected by RT-PCR in patients with AML by means of the IL-32θ-speicific primers: 5′-TGA TGT CGA GCC TGG CAG AG-3′ (sense) and 5′-GCT GCT CTC TGT CAG AGC TC-3′ (antisense). The mRNA expression level of IL-32γ was detected by quantitative real-time PCR in patients. The specific primers of IL-32γ were previously described [45]. The mRNA expression level of the proinflammatory cytokine TNF-α was evaluated by RT-PCR of total RNA samples extracted from THP-1 cells expressing either EV or IL-32θ after stimulation with PMA, LPS, and poly(I:C). TNF-α primer sequences were as follows: 5′-TAC ATC CTC GAC GGC ATC TCA-3′ (sense) and 5′-CTA CAT TTG CCG AAG AGC CCT-3′ (antisense).

### Assessment of cytokine production using an enzyme-linked immunosorbent assay (ELISA)

To determine whether IL-32θ can inhibit the expression of the proinflammatory cytokine TNF-α, IL-1β, and IL-6, we used biological samples from patients with AML as well as the culture supernatants from the THP-1 cells expressing IL-32θ (THP-1/IL-32θ). The cells were treated with several stimulators or inhibitors, such as PMA, LPS, poly(I:C), and rottlerin at the indicated dose and time points. After centrifugation, the culture media were collected and the expression levels for of the proinflammatory cytokines were quantified using the respective ELISA kits (R&D Systems, Minneapolis, MN) for each cytokine.

### TNF-α promoter activity assay

The vectors pGL3-TNF-α-promoter (1 μg) and pRL-null (Renilla, 0.1 μg) were cotransfected into THP-1 cells with or without the IL-32θ expression vector (1 μg) using the Neon™ transfection system (Invitrogen, Carlsbad, CA). Luciferase assays were performed using the Dual-Luciferase Reporter Assay System (Promega, Madison, WI).

### Immunoblotting assay

THP-1 and HL-60 cells were cotransfected with pcDNA3.1+-6xMyc-IL-32θ and then were incubated with 10 nM PMA. These cells were harvested and lysed in the 50 mM HEPES buffer (pH 7.5) containing 150 mM NaCl, 5% glycerol, 20 mM glycerophosphate, 1% Nonidet P-40, 0.5% Triton X-100, and 1 mM EDTA. Protein concentrations were determined using the BCA Protein Assay Kit (Bio-Rad), and the samples were subjected to 12% SDS-polyacrylamide gel electrophoresis (SDS-PAGE) before transferring to polyvinylidene difluoride (PVDF) membranes (Millipore, Billerica, MA). The membranes were blocked with 5% non-fat dry milk dissolved in PBST (140 mM NaCl, 27 mM KCl, 10 mM Na_2_HPO_4_ 12H_2_O, 1.8 mM KH2PO_4_, and 0.05% Tween-20). Horseradish peroxidase (HRP)-conjugated secondary antibodies were used to visualize the antigens using the Westzol plus Western Blot Detection System (iNtRON Biotechnology, Sungnam, Korea), and the data were processed using the EZ-capture MG protein imaging system (ATTO, Tokyo, Japan).

### Immunofluorescence assay

THP-1 cells were seeded on coverslips and cotransfected with 1 μg of pcDNA3.1+-6xMyc-IL-32θ. Then the cells were incubated with 10 nM PMA for 60 min. After the coverslips were isolated, the cells were fixed and permeabilized with 4% paraformaldehyde and 0.5% saponin for 10 min. After washing with PBS, we blocked the cells with 1% BSA, and then incubated them with primary antibodies (1:200 dilution) at room temperature for 2 h. After washing with PBS, we stained the cells with an anti-rabbit IgG antibody conjugated with fluorescein isothiocyanate (FITC; 1:400 dilution) for 2 h. After a wash with PBS, nuclei were stained with 4′,6-diamidino-2-phenylindole (DAPI; 1:2000 dilution) for 10 s. Images were acquired using an Olympus BX61–32FDIC upright fluorescence microscope (Olympus, Tokyo, Japan).

### The chromatin immunoprecipitation (ChIP) assay

THP-1/EV and THP-1/IL-32θ cells were incubated with PMA for 12 h. ChIP assays were carried out using the ChIP Assay Kit (Millipore) according to the manufacturer's instructions. Briefly, the cells were fixed with 1% formaldehyde for 10 min at 37°C, lysed for 10 min using 1 mL of the kit's lysis buffer with protease inhibitors, and then sonicated with 3 pulses for 10 s each. After the sonication, the samples were diluted 10-fold in ChIP Dilution Buffer, and centrifuged at 13,000 rpm for 15 min. The supernatants were incubated with or without the respective anti-NF-κB (p65) antibody at 4°C overnight, with rotation. Each sample was mixed with protein A-agarose/salmon sperm DNA (50% slurry), and the pulled down DNA fragments were eluted. The eluted DNA was amplified using PCR (35 cycles) at the annealing temperature of 58°C. The primer sequences for the NF-κB binding site in the TNF-α promoter were as follows: κB1, 5′-CAA GCA TTA TGA GTC TCC GG-3′ (sense) and 5′-AAG CTG TGT TGA GTC CTG AG-3′ (antisense); κB2, 5′-CCA GGG TCC TAC ACA CAA AT-3′ (sense) and 5′-CTC ATC TGG AGG AAG CGG TA-3′ (antisense); κB3, 5′-GCT TGT GTG TCC CCA ACT TT-3′ (sense) and 5′-GTG TGC CAA CAA CTG CCT TT-3′ (antisense).

### Statistical analysis

Statistical analyses involved one-way analysis of variance (ANOVA; with significance at *P* < 0.05) with Tukey's HSD test. The experiments contained 3 independent replicates and the data were expressed as mean ± standard deviation (SD, *n* = 3) with the following significance level: *P* < 0.05.
